# Identification of chemicals that mimic transcriptional changes associated with autism, brain aging and neurodegeneration

**DOI:** 10.1038/ncomms11173

**Published:** 2016-03-31

**Authors:** Brandon L. Pearson, Jeremy M. Simon, Eric S. McCoy, Gabriela Salazar, Giulia Fragola, Mark J. Zylka

**Affiliations:** 1 Department of Cell Biology and Physiology, UNC Neuroscience Center, University of North Carolina at Chapel Hill, 111 Mason Farm Road, Chapel Hill, North Carolina 27599-7545, USA; 2 Carolina Institute for Developmental Disabilities, University of North Carolina Chapel Hill, Chapel Hill, North Carolina 27599-7255, USA

## Abstract

Environmental factors, including pesticides, have been linked to autism and neurodegeneration risk using retrospective epidemiological studies. Here we sought to prospectively identify chemicals that share transcriptomic signatures with neurological disorders, by exposing mouse cortical neuron-enriched cultures to hundreds of chemicals commonly found in the environment and on food. We find that rotenone, a pesticide associated with Parkinson's disease risk, and certain fungicides, including pyraclostrobin, trifloxystrobin, famoxadone and fenamidone, produce transcriptional changes *in vitro* that are similar to those seen in brain samples from humans with autism, advanced age and neurodegeneration (Alzheimer's disease and Huntington's disease). These chemicals stimulate free radical production and disrupt microtubules in neurons, effects that can be reduced by pretreating with a microtubule stabilizer, an antioxidant, or with sulforaphane. Our study provides an approach to prospectively identify environmental chemicals that transcriptionally mimic autism and other brain disorders.

## Abstract (web summary)

This study presents gene expression responses of cultured brain cells to hundreds of chemicals found in the environment and in food. The authors identified chemicals that induce transcriptomic profiles that overlap those seen in human brains affected with autism, aging, and neurodegeneration.

## Graphical abstract

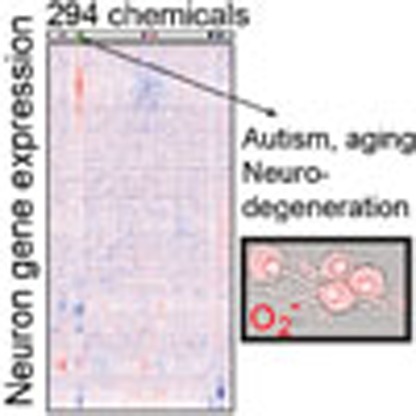

Powerful new sequencing technologies have been used to systematically identify hundreds of candidate gene mutations associated with autism spectrum disorder (ASD) risk^1^,^2^. Heritability studies suggest that environmental factors also contribute to autism risk^3^. Indeed, gestational exposure to pesticides, including maternal proximity to pesticide applications and runoff, is reproducibly associated with increased ASD risk in epidemiological studies^4^,^5^,^6^. However, epidemiological studies are retrospective and cannot ascertain prospectively, precisely or systematically which chemicals, of the >80,000 chemicals registered for use in the environment, have the greatest potential to harm the developing or adult brain^7^. Existing *in vivo* neurodevelopmental and neurotoxicological assays with animal models are labour intensive and costly, thus hindering throughput, whereas higher-throughput toxicological assays frequently use non-neuronal cells or focus on neuron death as an end point^8^,^9^. As a result, these tests fail to interrogate molecular and physiological processes that are unique to neurons or that differentiate normal from diseased human brains.

There is growing recognition that brain transcriptional changes are associated with ASD^10^,^11^. This ASD transcriptional signature is defined by reduced expression of genes involved in synaptic transmission and elevated expression of genes involved in immune and microglial function^10^,^11^. Here we hypothesized that this transcriptional signature might guide the prospective identification of candidate chemical risks for ASD. To test this hypothesis, we exposed mouse cortical neuron-enriched cultures to hundreds of environmental-use chemicals and then monitored global transcriptional changes. We identify six chemical groups, one of which mimics the transcriptional changes seen in ASD, but surprisingly also shares transcriptional similarity to the aged brain and certain neurodegenerative conditions. Our findings suggest these neurological conditions share a molecular pathology, as hypothesized by others^12^, despite different ages of onset and distinct behavioural symptoms. Moreover, our study shows that a transcriptional approach can be used to systematically scan a diverse chemical space and identify potential environmental threats to the human brain.

## Results

### Mouse cortical cultures transcriptionally model human brain

**Figure 1:**
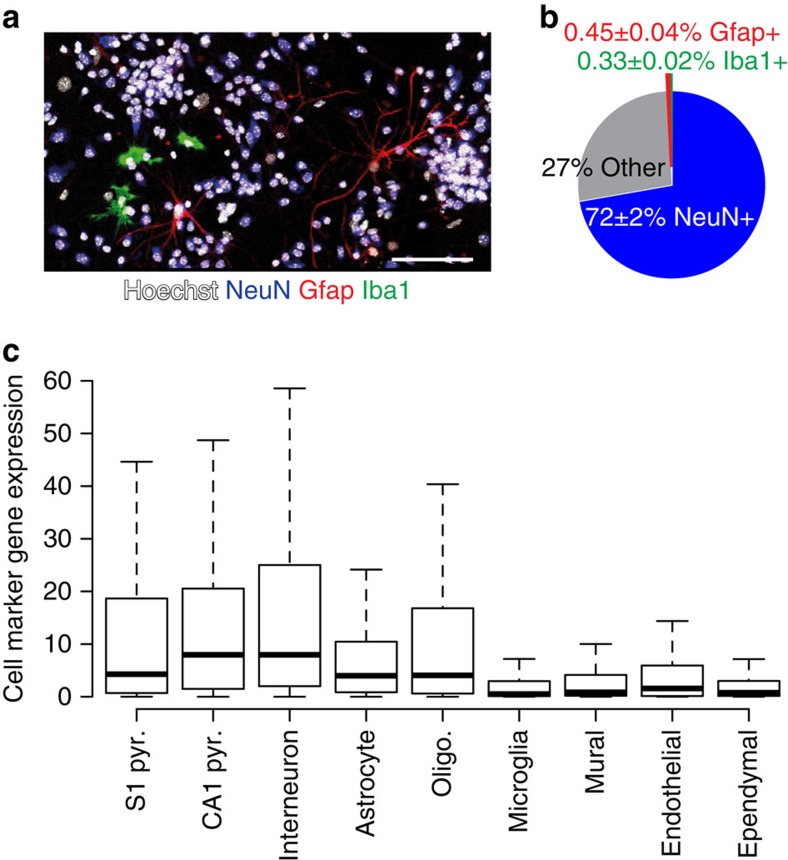
Mouse cortical cultures contain all principle brain cell types. (**a**–**b**) Vehicle-treated cortical cultures contain NeuN-positive neurons, Gfap-positive astrocytes and Iba1-positive microglia. *n*=98,890–145,032 cells per coverslip counted. Values are mean±s.e.m. of four biological replicates. Scale bar, 100 μm. (**c**) Cortical cultures exhibit expression of marker genes representative of nine cell types found in the brain (*y* axis RPKM).

To determine whether mouse cortical cultures model cellular and molecular aspects of the human brain, we performed immunocytochemistry with cellular markers and compared the gene expression profile of our cultures with brain cell-type-specific expression data sets and human brain gene expression data sets, including the Allen BrainSpan atlas (www.brainspan.org) and GTEx^13^,^14^. Our cultures contained many of the principle cell types of the brain based on immunocytochemistry with markers for neurons, astrocytes and microglia (∼25% of cultures were non-neuronal cells; Fig. 1a,b). In addition, the expression of markers for each brain cell type^13^ in our cultures was highly correlated with that of whole embryonic (E14.5) brain (Pearson *r*=0.8), further suggesting that our culture system represented all major cell classes in biologically realistic proportions (Fig. 1c; Supplementary Fig. 1a,b). Globally, gene expression in our cortical cultures correlated more strongly with each human brain region (except spinal cord) than to any other tissues of the human body (Supplementary Fig. 2a). The strongest correlations were to frontal cortex and anterior cingulate cortex, regions implicated in ASD^3^. Moreover, cultures were most strongly correlated with frontal cortex from mid-late gestation human brain (Supplementary Fig. 2b), the developmental time window implicated in ASD pathogenesis^15^. In contrast, terminally differentiated neurons derived from human embryonic stem cells^16^ did not strongly correlate with any brain region, and instead were most similar to tissues associated with the female reproductive system (Supplementary Fig. 2c). Taken together, our cortical cultures show strong transcriptional similarities to the human brain.

### Transcriptional screen with environmental chemicals

We next measured cytotoxicity of the 294 chemicals in the US Environmental Protection Agency (EPA) ToxCast Phase I library, which includes common food-use pesticides and other environmental chemicals, such as plasticizers^8^, to identify a non-toxic concentration for RNA sequencing (RNA-seq). We treated cultures in quadruplicate for 24 h at 10 μM, as this is a common concentration used in screening studies^17^, then used fluorescent markers to quantify the proportion of live and dead cells (Supplementary Fig. 3). Most (87%) of the chemicals were not cytotoxic at 10 μM, whereas concentrations of the remaining chemicals had to be reduced to between 10 and 100 nM (Supplementary Data 1). Fresh cultures were then treated (24 h) with each chemical at the non-toxic concentration or with vehicle (equivalent dimethylsulphoxide (DMSO) concentration ≤0.5%), as the negative control. We also tested three topoisomerase 1 (TOP1) inhibitors, as they reproducibly downregulate long (>100 kb) genes^18^.

**Figure 2:**
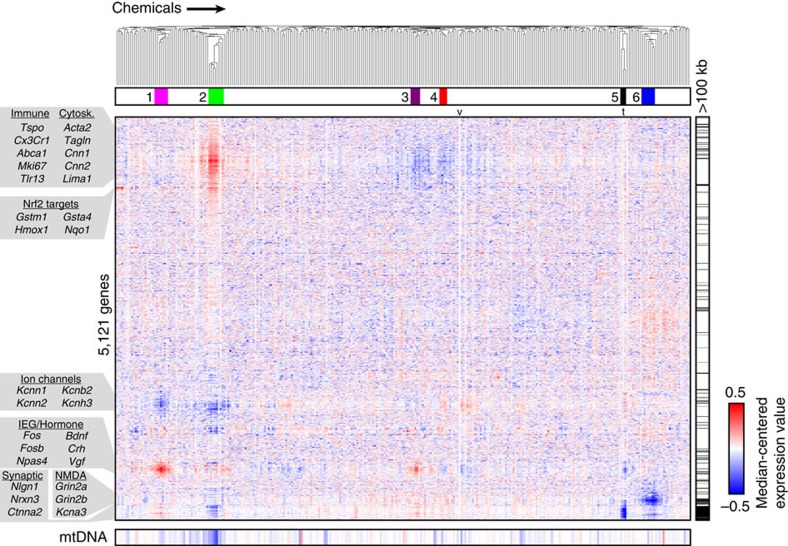
Gene expression defines six chemical clusters in cortical neuron cultures. Median-centred gene expression values for 297 chemicals and vehicle (v; median of 49 replicates, t; topotecan-positive control, median of 32 replicates) were hierarchically clustered across 5,121 variably expressed genes. Genes >100 kb in length are tick marked (right), and mitochondrial health (bottom) was estimated by comparing the fraction of reads that align to the mitochondrial genome for each chemical–vehicle pair (blue indicates negative log_2_ fold change with respect to vehicle). mtDNA, mitochondrial DNA.

To identify chemicals that caused concordant gene expression changes, we performed hierarchical clustering of 5,121 genes variably expressed across all chemicals, and six chemical groups emerged (hereafter referred to as clusters 1–6; Fig. 2; see Methods and Supplementary Fig. 4 for batch correction details). Individual chemicals were assigned to a cluster using a pairwise correlation threshold and were validated to be statistically and biologically significant (Supplementary Fig. 5; see Methods for further details on inclusion/exclusion criteria). To evaluate whether existing *in vitro* ToxCast assay data could identify similar chemical relationships, we performed clustering analysis across 199 cell-free and cellular (non-neuronal) assays. The resulting chemical groupings did not resemble those we detected by gene expression profiling (Supplementary Fig. 6). Thus, transcriptional profiling with neuronal cells identifies relationships between chemicals that existing toxicological assays failed to detect.

To determine what these functional relationships were, we examined specific genes with altered expression in each cluster (Fig. 2). Cluster 1 chemicals upregulated immediate early genes (IEGs) while reducing expression of several potassium channel genes. These expression changes were suggestive of neuronal hyperexcitability. Supporting this possibility, cluster 1 included two pyrethroids (cyfluthrin and fenpropathrin) that stimulate sustained calcium influx in cortical neurons^19^. Cluster 2 chemicals upregulated numerous immune and cytoskeletal transcripts but reduced expression of ion channel and synaptic genes. Cluster 3 chemicals upregulated IEGs but reduced the expression of immune and cytoskeletal genes. Cluster 4 chemicals resembled cluster 3, but without inducing IEGs. Cluster 5 contained all three TOP1 inhibitors, which markedly downregulated long genes and downregulated IEGs, consistent with one of these inhibitors (topotecan) reducing spontaneous synaptic activity in cortical neuron cultures^20^. Deletion of a related topoisomerase (TOP2B) reduced IEG expression^21^. Cluster 6 chemicals reduced expression of several neurotransmitter receptor subunits, including *N*-methyl-D-aspartate receptor subunits (*Grin2a* and *Grin2b*), cholinergic receptors (*Chrm2* and *Chrna7*) and ion channels.

### Cluster 2 transcriptionally mimics specific brain disorders

**Figure 3:**
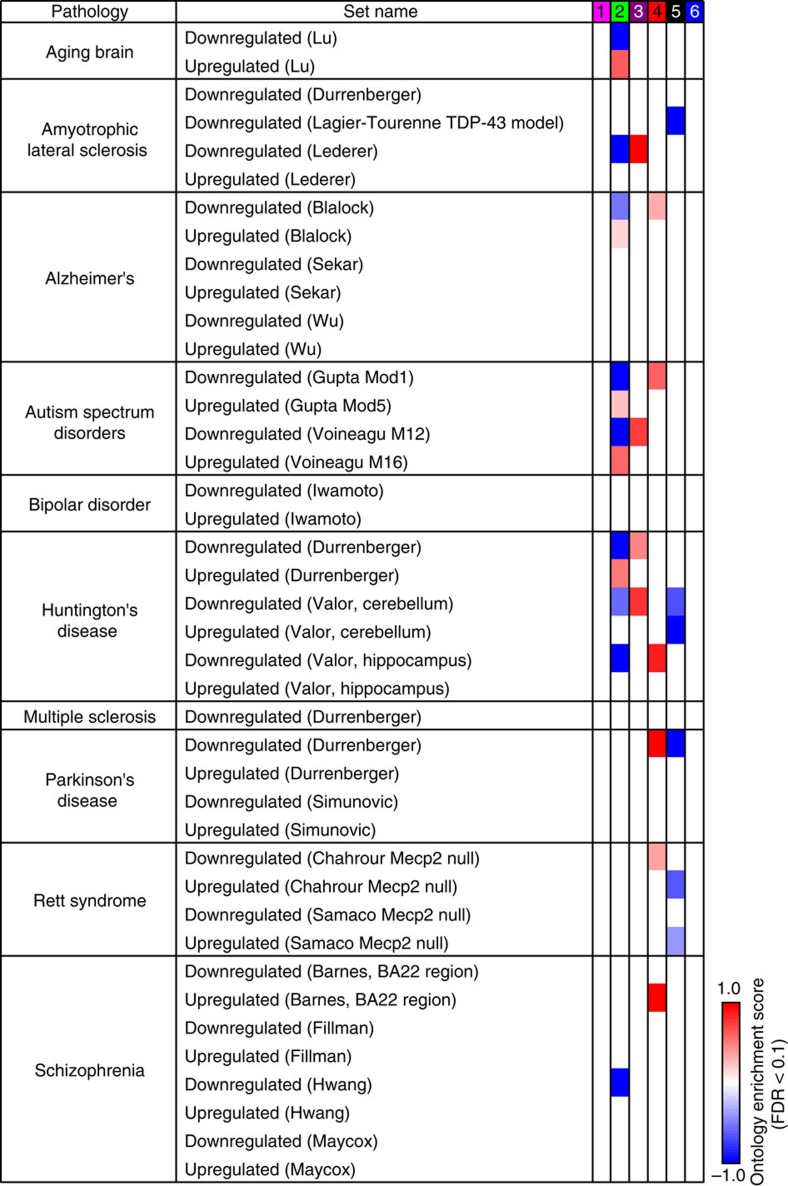
Cluster 2 chemicals show significant gene set enrichment with autism and other brain diseases. The enrichment scores of brain disease gene sets that were statistically significant (FDR<0.1) in each chemical cluster were plotted on a scale from −1 (blue) to +1 (red). Author surname and/or gene set name are indicated in parentheses. The composition of these gene sets is shown in Supplementary Data 2.

We next identified the molecular pathways that were differentially associated with the six chemical clusters using gene set analysis (GSA). Included in this analysis were 64 gene sets for human brain disorders and nervous system pathways that we generated from publications (Supplementary Data 2) alongside 4,722 curated pathways. Over 600 gene sets distinguished the six clusters (Supplementary Fig. 7; Supplementary Data 3), including many of the human brain disorder gene sets (Fig. 3). However, only cluster 2 chemicals mimicked the transcriptional changes of two post-mortem ASD brain expression data sets in a bidirectional manner. This included downregulation of M12 and Mod1 (largely composed of synaptic genes that are downregulated in ASD brains), and upregulation of M16 and Mod5 (largely composed of microglial/immune genes that are upregulated in ASD brains)^10^,^11^. In addition, cluster 2 showed concordance with aging human brain and two neurodegenerative disorders (Alzheimer's disease and Huntington's disease), suggesting shared aspects of molecular pathology despite different symptoms (see Discussion).

**Figure 4:**
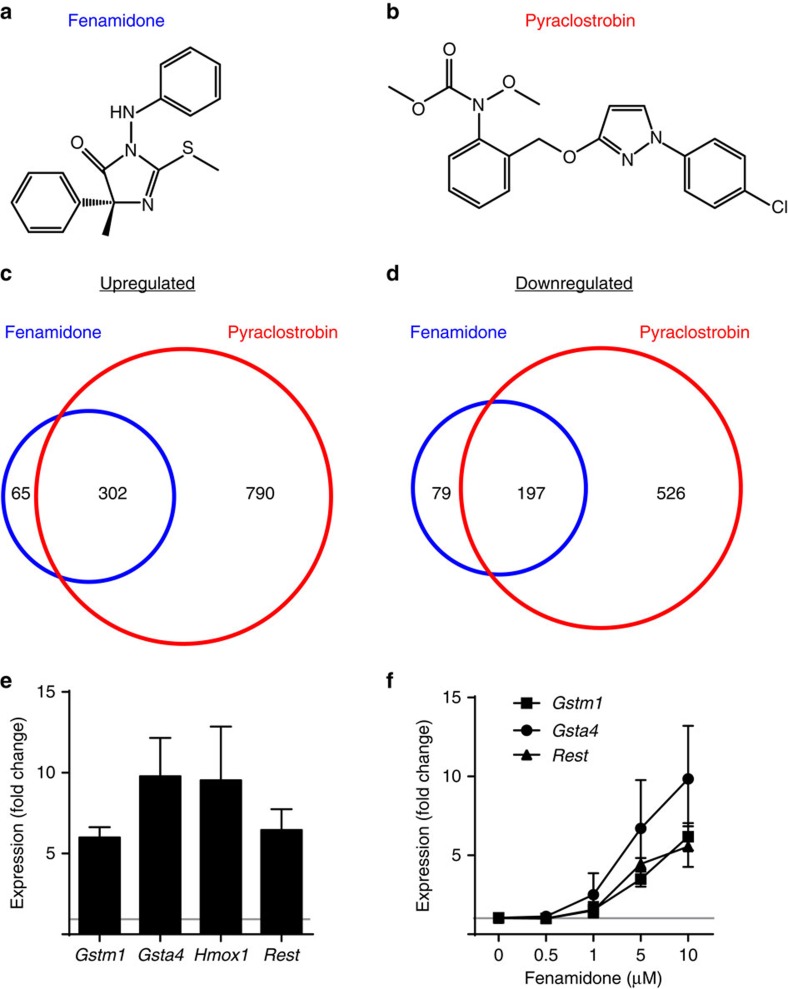
Cluster 2 chemicals alter expression of a common set of genes. (**a**) Fenamidone and (**b**) pyraclostrobin (**c**,**d**) up- and downregulate a largely overlapping set of genes. RNA-seq was performed in quadruplicate after treating cortical cultures with fenamidone (10 μM) or pyraclostrobin (0.1 μM) for 24 h (*n*=3 biological replicates) and compared with matched vehicle controls using DESeq to detect differentially expressed genes, listed in Supplementary Data 4. Quantitative RT–PCR fold change (mean±s.e.m. across three to four biological replicates) of selected cluster 2 upregulated genes relative to vehicle after 24 h treatment with (**e**) 10 μM fenamidone or (**f**) the indicated doses of fenamidone.

Fenamidone, pyraclostrobin and two other chemicals in cluster 2 (famoxadone and trifloxystrobin) are members of a recently developed class of fungicides that inhibit mitochondrial complex III by targeting the quinone outside (Qo) site of cytochrome *bc*_1_ (ref. 22). Since fenamidone and pyraclostrobin are structurally distinct (Fig. 4a,b), we performed RNA-seq with multiple replicates of both the chemicals to further validate that expression of a common set of genes was altered. Each fungicide altered expression of a largely overlapping set of genes (Fig. 4c,d; Supplementary Data 4). Upregulated transcripts included *Nrf2* target antioxidant response genes and *Rest*, which we validated using quantitative real-time PCR (qRT–PCR) (Fig. 4e,f). *Rest* elevation is associated with human brain aging and neurodegeneration^23^. Other compounds in cluster 2 include fenpyroximate, pyridaben and rotenone, chemicals that target mitochondrial complex I (ref. 24). Exposure to rotenone is known to increase risk for Parkinson's disease^24^,^25^. All cluster 2 chemicals reduced RNA-seq reads arising from the mitochondrial genome (Fig. 2), suggesting compromised mitochondrial function as a common mechanism^26^. Note that none of the mitochondrially encoded transcripts were among the 5,121 variably expressed genes assayed by hierarchical clustering, so these transcripts did not influence cluster assignment. However, the influence of chemical treatment on mitochondrial density or viability was not assessed and could have indirectly modulated transcriptional profiles.

### Cluster 2 induces superoxide and microtubule instability

**Figure 5:**
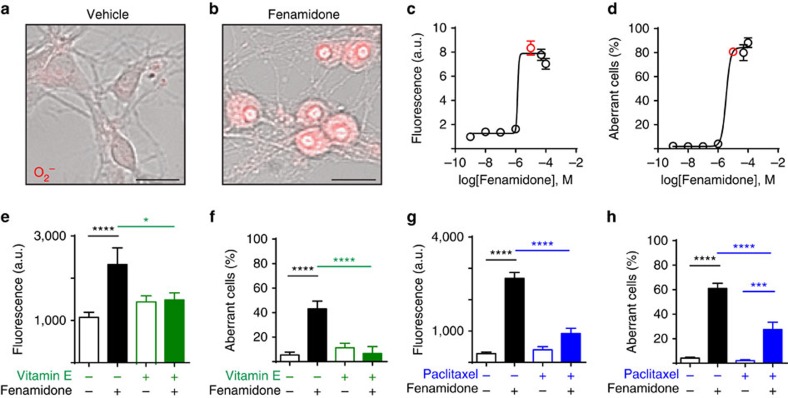
Fenamidone causes mitochondrial superoxide production and microtubule destabilization. (**a**,**b**) Superoxide (O_2_^−^, MitoSOX fluorescent indicator) and aberrant cell morphology elicited by 2-h treatment with 10 μM fenamidone. Scale bars, 10 μm. (**c**) O_2_^−^ generation and (**d**) aberrant morphology is dose dependent. RNA-seq dose is denoted by red circles. (**e**) Pretreatment with vitamin E (10 μM, 2 h) blocked O_2_^−^ formation and (**f**) aberrant morphology elicited by fenamidone (10 μM, 2 h). (**g**) Microtubule stabilization with paclitaxel pretreatment (10 μM, 2 h) attenuated O_2_^−^ formation and (**h**) aberrant morphology elicited by fenamidone (10 μM, 2 h). All values mean±s.e.m. *n*=33–256 cells per condition across six biological replicates. Bar graphs display mean±s.e.m. *P<0.05, ***P<0.001, ****P<0.0001.

**Figure 6:**
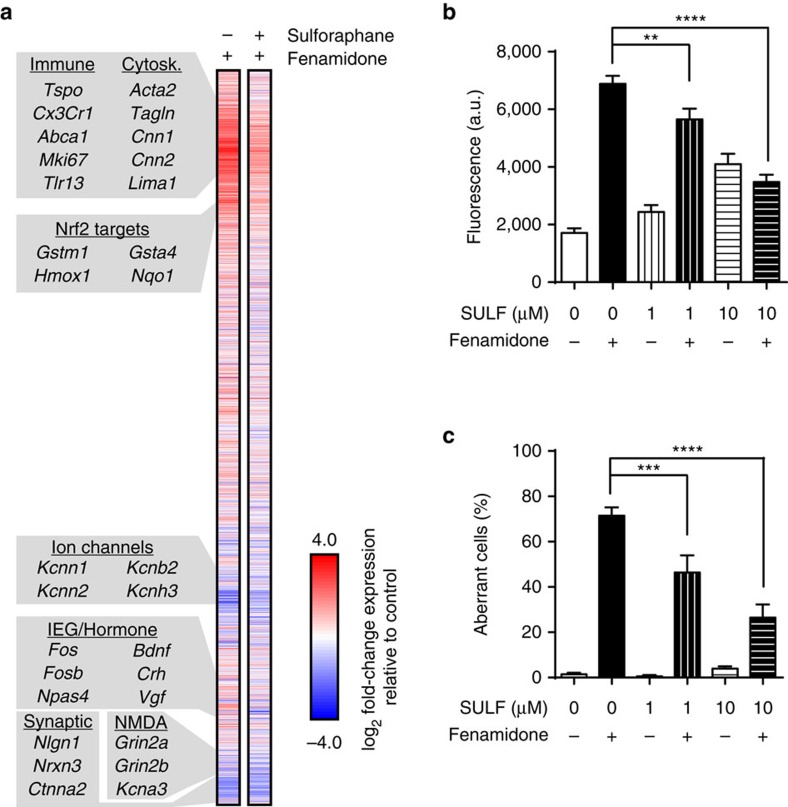
Sulforaphane attenuated fenamidone-induced transcriptional and cellular responses in cortical cultures. (**a**) Genome-wide (RNA-seq) transcriptional changes caused by fenamidone (10 μM, 24 h; *n*=3 replicates) and fenamidone (10 μM, 24 h) after pretreating with sulforaphane (10 μM, 18 h; *n*=3 replicates). Gene order is identical to Fig. 2. (**b**) Fenamidone-induced (10 μM, 2 h) O_2_^−^ production and (**c**) aberrant cell morphology were attenuated by pretreating (18 h) with two concentrations of sulforaphane (SULF). *n*=175–327 cells per condition across 6–18 biological replicates. Bar graphs display Mean±s.e.m. **P<0.01, ***P<0.001, ****P<0.0001.

Mitochondrial complexes I and III are the main sites of superoxide (O_2_^−^) production within the electron transport chain, so we next tested whether cluster 2 chemicals induced O_2_^−^. Fenamidone produced a concentration-dependent increase in O_2_^−^, as measured with a fluorescent mitochondrial superoxide indicator dye, and swelling of neuronal soma (Fig. 5a–d). Pretreatment with the free-radical scavenger vitamin E (α-tocopherol) blocked fenamidone-induced production of O_2_^−^ (Fig. 5e; using the concentration of fenamidone that was used for sequencing) and blocked soma swelling (Fig. 5f). Sulforaphane is a potent inducer of *Nrf2* target antioxidant gene expression, it reduces inflammation and it substantially reduces autism symptoms in humans^27^,^28^. Pretreatment with sulforaphane attenuated the transcriptional changes, O_2_^−^ production and soma swelling caused by fenamidone (Fig. 6). We also monitored O_2_^−^ production for each chemical in cluster 2 and for several chemicals outside of cluster 2 across a seven-point concentration–response range (Supplementary Fig. 8). All chemicals that induced O_2_^−^ at the sequencing concentration were assigned to cluster 2 based on gene expression. Fluoxastrobin and azoxystrobin are Qo fungicides that were not assigned to cluster 2, likely because they did not generate O_2_^−^ at the sequencing concentration (Supplementary Fig. 8). Two additional mitochondrial inhibitors that induce O_2_^−^ production in cells (myxothiazol, complex III inhibitor; kresoxim-methyl, complex III inhibitor and fungicide) altered transcription in a manner consistent with chemicals in cluster 2 (Supplementary Fig. 9). Collectively, our data support a relationship between O_2_^−^ production and the cluster 2 transcriptional signature.

Numerous genes associated with cytoskeletal function have been implicated in autism^29^. Moreover, disruption of microtubules is associated with problems in brain development and with neurodegeneration^30^,^31^. Given that cytoskeletal genes were altered by cluster 2 chemicals and that rotenone (also in cluster 2) destabilizes microtubules^31^,^32^, we hypothesized that microtubule depolymerization triggered the aberrant swelling morphology of neurons. Consistent with this possibility, stabilization of microtubules with paclitaxel attenuated fenamidone-induced O_2_^−^ production and fenamidone-induced soma swelling (Fig. 5g,h). However, fenamidone did not impair tubulin polymerization in a cell-free biochemical assay, ruling out a direct effect of fenamidone on tubulin oligomerization (Supplementary Fig. 10a). Last, superoxide production and soma swelling could be phenocopied by depolymerizing microtubules with vincristine (Supplementary Fig. 10b–e).

### Assessment of potential confounds

Cluster 2 compounds upregulated numerous microglial- (for example, *Cx3cr1* and *Trem2*) and astrocyte- (for example, *Gfap* and *Aqp4*) enriched genes^33^. This increase in markers of proliferating brain cell types was unlikely to be due to cell division, as our cultures were treated with an antimitotic on days *in vitro* (DIV) 3. Moreover, fenamidone (under the same conditions used for sequencing) caused no significant change in cellular composition, based on immunocytochemical quantification of neurons, astrocytes and microglia with markers (NeuN, Gfap and Iba1, respectively; Supplementary Fig. 11a). However, any contribution by additional non-neuronal cell types such as oligodendrocytes has not been accounted for. Neither culture batch nor sequencing batch effects contributed to cluster identity (Supplementary Fig. 4). We also assessed RNA quality using the RNA integrity number (RIN) as a proxy. There were subtle differences in quality among the six clusters, and RINs were significantly lower for clusters 2 and 6 (Supplementary Fig. 11b). However, the average RINs were ≥9.25 for each of the six clusters, indicating that these samples were of sufficiently high quality. Collectively, we found no evidence that technical artefacts or poor RNA quality contributed to cluster identity. However, we cannot exclude the possibility that some of the chemicals alter cellular composition at higher concentrations or after longer exposure times.

### Increasing agricultural use of several cluster 2 chemicals

**Figure 7:**
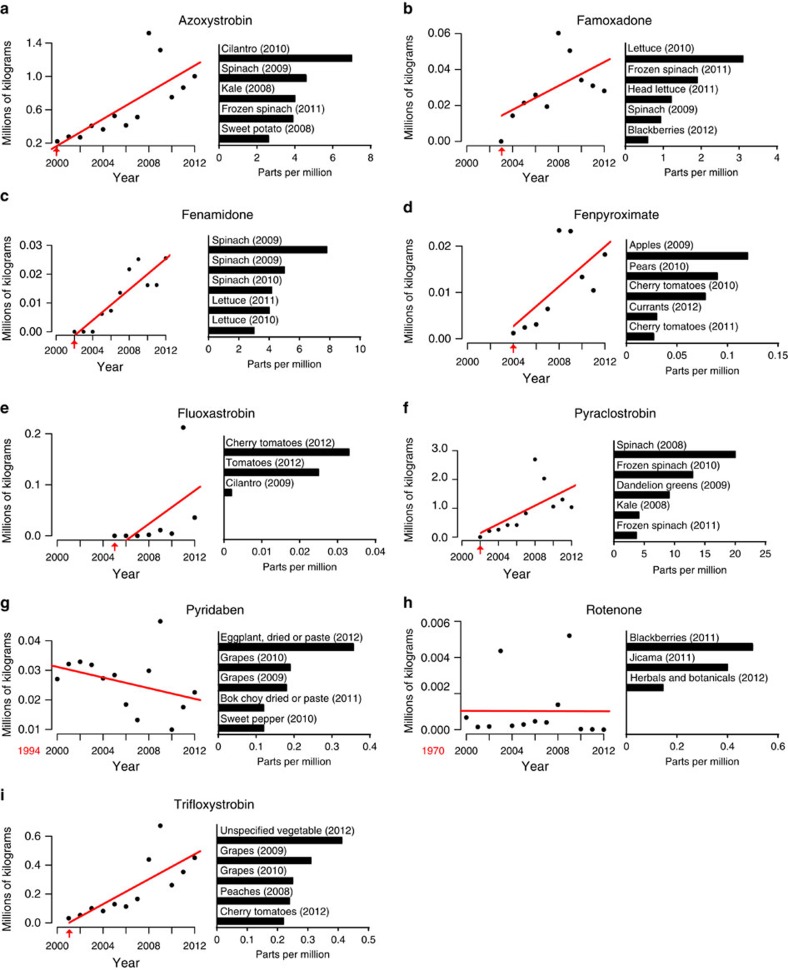
Usage trends and environmental fate of cluster 2 chemicals. (**a**–**i**) Left: amount of chemical (alphabetical order) applied in the United States based on United States Geological Survey data. (**a**–**i**) Right: the five foods with the highest residue levels and the year of detection based on USDA and FDA data spanning 2008–2012. Red arrows indicate the year each chemical was first registered for use with the EPA. Chemicals approved before 2000 list the registration year in red font below the *x* axis. Spinach tested for high levels of fenamidone in both the 2009 USDA and FDA surveys (**c**), hence explaining why ‘Spinach (2009)' is displayed twice.

Epidemiological and human exposure data for most chemicals in cluster 2 are lacking. We thus sought to evaluate exposure potential by analysing chemical usage and food commodity residue data collected by the United States Geological Survey, the United States Department of Agriculture (USDA) and the Food and Drug Administration (FDA). All of the mitochondrial complex III inhibitors in cluster 2 showed positive environmental usage trends since their EPA registration in 2000 or later (Fig. 7). Usage of complex I inhibitors (rotenone and pyridaben) is low and unchanging, with the notable exception of fenpyroximate, the most potent superoxide producer we identified (concentration for half-maximum response (EC50)=0.007 μM; Supplementary Fig. 8b). Many cluster 2 residues were found on conventionally raised food commodities, particularly leafy green vegetables, and were detected at relatively high levels, up to 20 p.p.m. in the case of pyraclostrobin. These data suggest significant human exposure potential to many of the chemicals in cluster 2.

## Discussion

By comparing gene expression profiles of cortical cell cultures with expression data from human brain disorders, we identified a group of eight chemicals (cluster 2) that transcriptionally mimicked ASD, brain aging and neurodegeneration. These chemicals, most of which inhibit mitochondrial complex I or III, stimulated free radical production and disrupted microtubules. We found that pretreating with a microtubule stabilizer, an antioxidant, or with sulforaphane could reduce these effects. Whether this transcriptional and cellular response is related to the marked clinical efficacy of sulforaphane at treating ASD symptoms^27^ remains to be determined.

Numerous studies investigated a link between the inhibition of mitochondrial complex I, neurotoxicity and neurodegeneration in animal models^24^,^34^,^35^. Rotenone (in cluster 2) has been shown to increase Parkinson's disease risk in humans^25^. Cluster 2 also included a relatively new class of fungicides (quinone outside, Qo) that inhibit mitochondrial complex III. No evidence of neurotoxicity was noted for two of these fungicides, pyraclostrobin and fenamidone, in a set of assays used by regulatory agencies^36^,^37^. However, a single oral dose of trifloxystrobin (also in cluster 2) reduced motor activity for several hours in female rats and for 3 days in males^38^, suggesting a strong interaction with sex. Picoxystrobin (not in ToxCast Phase I library), marketed as the most rapidly absorbed and most systemic (in plants) of all Qo fungicides, caused acute neurotoxicity (reduced motor activity) at the lowest dose tested in rats^39^. Further, mitochondrial complex III-deficient mice showed severe superoxide-dependent damage to cortical brain regions and profound motor deficits that were apparent at night, during their active phase^40^. Note that standard acute and chronic neurotoxicity assays are performed during the day, when rodents are less active, possibly reducing the power to detect motor deficits. Mitochondrial complex III inhibitors can additionally block neuronal differentiation by maintaining embryonic stem cell pluripotency^41^, suggesting a potential for neurodevelopmental effects.

Usage data indicate that Qo fungicides are increasingly prevalent on food that is consumed by humans of all ages. At least one cluster 2 chemical (pyraclostrobin) is present in the environment at levels that affect non-mammalian organisms^42^,^43^ and was detected at high levels on foraging honeybees, further corroborating high levels in the environment^42^. To address whether these levels are a risk to human health, recent *in vitro* reverse dosimetry extrapolations from the EPA found that food levels of pyraclostrobin exceed the human oral equivalent dose necessary to affect mitochondrial processes^44^. However, Qo fungicide residues have not been detected on organically produced foods (EPA and USDA data), suggesting a way to minimize exposure.

Our finding that cluster 2 chemicals mimic the transcriptional changes of autism, as well as the aging brain and neurodegeneration was surprising, particularly given the different ages of onset and disease symptoms. Oxidative stress and cytoskeletal integrity are implicated in all of these conditions^45^,^46^,^47^,^48^,^49^, suggesting that overlapping pathological processes might drive the transcriptional similarities we observed. In support of shared biology, we found that ASD, the aging brain, Alzheimer's disease and Huntington's disease exhibit altered expression of a common set of genes more so than any of the other neurological gene sets we tested (Supplementary Fig. 12a). Many of the genes that were differentially regulated by pyraclostrobin were also found in the M16 and M12 ASD gene modules (Supplementary Fig. 12b–e). Moreover, when focusing specifically on the genes in these ASD modules, the direction and magnitude by which these genes were dysregulated in ASD patient samples was strongly correlated with that of pyraclostrobin treatment in our cortical cultures (Spearman *r*=0.66; Supplementary Fig. 12f), further suggesting a common mechanism. The fact that these neurological conditions shared a core set of dysregulated genes may contribute to the enrichment observed for cluster 2 across these diseases. However, we cannot exclude the possibility that molecular pathologies are shared by some but not all of the conditions. Disentangling these relationships is beyond the scope of our current study, but suggests a fruitful area for future research.

Several of the chemicals in cluster 2 unquestionably kill neurons at higher concentrations (Supplementary Data 1), consistent with other studies^9^,^50^. This raises the question of whether cluster 2 reflects the transcriptional signature of ‘sick' neurons. We identified several chemicals that killed cells at multiple concentrations (Supplementary Data 1), yet only those that were associated with O_2_^−^ production, microtubule destabilization and elevated expression of neuroinflammatory genes were assigned to cluster 2. It thus seems unlikely that cluster 2 is reflective of chemicals that nonspecifically kill neurons at high doses and sicken neurons at lower doses. In fact, chemicals can kill (and presumably sicken) cells via distinct mechanisms^32^, with one of these mechanisms being ‘microtubule destabilization.' Rotenone and vincristine fit within this class^32^, likely providing additional insights into why paclitaxel (a microtubule stabilizer) attenuated the soma swelling and O_2_^−^ production phenotypes induced by a cluster 2 chemical (Fig. 5). Our study also shows how systematic transcriptional studies with neurons can uncover new brain- and disease-relevant relationships between chemicals that cannot be identified using existing toxicology assays (Supplementary Fig. 6), including those that rely on cell death as a readout.

We identified additional brain-relevant relationships between chemicals within other clusters. Cluster 1 appears to define a transcriptional signature of neuron hyperexcitability, as evidenced by upregulation of IEGs, a class of genes that mark recently depolarized neurons, and downregulation of potassium channels (which increases neuron excitability when downregulated^51^). Cluster 1 contained two pyrethroids that hyperexcite mammalian neurons^19^. Intriguingly, two recent epidemiological studies found that pyrethroid exposure doubles the risk for attention deficit hyperactivity disorder in boys^52^,^53^. Our transcriptional approach might provide a way to prospectively identify candidate chemical risks for attention deficit hyperactivity disorder. Cluster 5 contained all three topoisomerase inhibitors, a class of drugs that downregulated long genes in neurons^18^, reduced synaptic activity^20^ and reduced IEG expression (Fig. 2). Moreover, cluster 5 is strongly correlated with neurological disease models that feature dysregulated long synaptic gene expression, particularly amyotrophic lateral sclerosis^54^ and Rett syndrome^55^,^56^.

Though estimates vary, ∼50% of cells in the adult human central nervous system are neurons^57^. This is in contrast to the embryonic culture system employed here, which is comprised of over 70% neurons. This disparity has the potential to bias physiological signatures and impair the ability to detect some disease-relevant processes. Although our cultures show a very similar representation of brain cell markers relative to E14.5 whole mouse brain (Supplementary Fig. 1), suggesting our cortical cultures—dissected at E14.5—contain the major brain cell classes in biologically realistic proportions. Moreover, the model system employed here is amenable to high-throughput screens and functional assays^17^. Ultimately, candidate chemicals identified with a cortical culture system will require validation in animal models.

In summary, our study shows that chemicals that transcriptionally mimic brain disorders can be identified by profiling gene expression in cortical cultures. This approach may also prove useful in identifying candidate ASD therapeutics, such as sulforaphane, or in identifying drugs that normalize long gene expression, such as topoisomerase inhibitors in Rett syndrome model neurons^55^. While usage and residue levels of cluster 2 chemicals on conventionally grown foods are increasing, in the absence of causality, it is premature to draw correlations with the increased prevalence of ASD and other brain disorders. Nonetheless, greater scrutiny over whether these new Qo fungicides affect the developing or adult mammalian nervous system (enteric, peripheral and central) or behaviours seems warranted, particularly given their striking mechanistic similarity to rotenone. Ultimately, monitoring steady-state levels in the environment, assessing exposure levels and pharmacokinetics, and epidemiological studies will be needed to evaluate whether any of these chemicals pose real neurological threats to humans or increase risk for brain disorders, including ASD.

## Methods

### Cortical neuron culture

Primary mouse cortical neuron cultures were prepared as previously described from E14.5 pregnant C57BL/6J (Cat. #000664, Jackson) dams crossed to CAST/EiJ (Cat. #000928, Jackson) males^18^. Hybrid cultures are thought to better model genetic variation associated with human populations^58^. Dissociated cells were placed in multiwell plates coated with poly-D-lysine (0.1 mg ml^−1^) in Neurobasal medium (Life Technologies) containing 5% fetal bovine serum (Gibco), B27 (17504-044, Invitrogen), Antibiotic-Antimycotic (15240-062, Invitrogen) and GlutaMAX (35050-061, Invitrogen). At DIV 3, a half medium change was performed with feeding medium identical to the plating medium except that we omitted fetal bovine serum and included 4.84 μg ml^−1^ uridine 5′-triphosphate (U6625, Sigma-Aldrich) and 2.46 μg ml^−1^ 5-fluoro-2′-deoxyuridine (F0503, Sigma-Aldrich) to inhibit mitosis in dividing cells.

### Chemicals

The chemical library was donated by the EPA ToxCast Program. For verification and replication experiments, the following chemicals were purchased from Sigma-Aldrich: DL-α tocopherol acetate (T3376), DL-sulforophane (S4441), vincristine sulfate salt (V8879), oxyfluorfen (35031), rotenone (45656), fenamidone (33965), pyraclostrobin (33696), trifloxystrobin (46447), myxothiazol (T5580), pyridaben (46047), azoxystrobin (31697), fluoxastrobin (33797), fenpyroximate (31684) and kresoxim-methyl (37899). Famoxadone was purchased from Chem Service, Inc (N-11943). Topotecan hydrochloride was purchased from Tocris (4562). Paclitaxel was purchased from Fisher Scientific (AC32842). All chemical stocks were prepared in DMSO unless otherwise noted. Vehicle samples were prepared with an equivalent DMSO concentration of ≤0.5% in feeding medium.

### Live/dead assay

Cultures were grown on poly-D-lysine coated 96-well plates at a density of 5 × 10^4^ cells per well. Culture plates were screened for quality by qualitative assessment of density and by confirming low percentage (<10%) of dead cells in control wells as established by the method described below. Chemicals were applied in quadruplicate by performing a half medium change with a 2 × concentration in prewarmed feeding medium on culture plates meeting these quality criteria, at DIV 7. After 24 h, another half medium change was performed with medium containing NucBlue (R37605, Life Technologies) and SYTOX Green (S7020, Life Technologies) dyes according to the manufacturer's directions. Cells were then rinsed twice with PBS (137 mM NaCl, 10 mM Na_2_HPO_4_, 1.8 mM KH_2_PO_4_) and fixed with 4% paraformaldehyde in 0.1 M phosphate buffer for 10 min at room temperature. After rinsing an additional two times with PBS, the cells were imaged on a Nikon Eclipse Ti epifluorescent microscope at × 10 magnification with ultraviolet excitation to reveal the NucBlue dye marking all cells, and with fluorescein isothiocyanate filter set to identify dead SYTOX-positive cells. Channel-merged colour images were imported into ImageJ software (NIH) and converted into RGB stacks. A manual threshold was performed on the NucBlue channel to yield an image amenable to binary particle analysis. The threshold set for the NucBlue channel was utilized for the SYTOX channel to reduce any potential bias. Particle analysis was performed on each channel for each image to obtain the total number of nuclei and the total number of nuclei from dead cells, respectively. The percentage of dead cells was calculated and averaged across four replicates. All chemicals from the library were initially tested at 10 μM. A concentration causing 10% or greater cell death relative to vehicle was considered toxic. Lower doses were tested by reducing an order of magnitude until a non-toxic dose was identified.

### RNA-seq

Cultures were treated with the non-cytotoxic dose of each chemical, by exchanging half of the original medium with chemicals at 2 × concentration in prewarmed feeding medium, on DIV 7 for 24 h in 12-well plates at a density of 5 × 10^5^ cells per well. RNA was isolated using RNeasy plus mini kit (Cat. #74134, Qiagen). RNA yield and quality were determined using a Nanodrop 1000 Spectrophotometer (Thermo Scientific). Samples were further assessed for quality using either an Agilent Bioanalyzer 2100 or TapeStation 2200 to obtain a RIN. RIN values exceeding 7 were used for sequencing. RNA samples were used to generate and barcode complementary DNA libraries using the TruSeq RNA Library Preparation Kit at the UNC High Throughput Sequencing Facility. Pools of 24 multiplexed samples were sequenced per lane on an Illumina HiSeq 2500 using 50-bp paired-end reads.

### RNA-seq data processing

RNA-seq reads were filtered using TagDust and aligned to the reference mouse genome (mm9) with TopHat using default parameters^59^,^60^. Reads aligning to ribosomal RNA genes were removed. Transcript abundance was estimated by computing Reads per kilobase per million mapped reads (RPKM) using RefSeq gene models aggregated by gene symbol^61^. For differential expression analyses, raw counts over RefSeq exons were used, and then were compared across samples using DESeq^62^.

### Hierarchical clustering and cluster membership

RPKMs for all RefSeq genes and all samples were filtered such that a given gene had >90% of samples with RPKM >0 and gene length exceeded 500 bp. RPKMs were then log_10_-transformed, quantile-normalized, median-centred, and the effects due to culture or sequencing batch were removed using a mixed model analysis of variance. This batch correction step was performed on individual experiments rather than chemical–vehicle ratios to reduce the effects due to variations in culture composition as well as batch-to-batch variations at the level of sequencing. Genes were then median-centred again, and filtered such that their s.d. exceeded 0.1. Chemical replicates were then combined using the median. Classes of chemicals were then discovered in two ways. First, we performed hierarchical clustering using average linkage and Pearson correlation distance measures. The boundaries of the discovered classes from clustering were set by computing pairwise Spearman correlations for all samples. A given cluster had to have at least three members with the minimum pairwise Spearman correlation coefficient exceeding 0.2, though cluster members often exceeded a correlation of 0.6 (Supplementary Fig. 5c,d). Seven chemical clusters were discovered, however one was removed due to failing to have a positive silhouette width, a metric of cluster robustness^63^. These definitions enabled the three positive control topoisomerase inhibitors to form a cohesive group of chemicals with known structural and functional similarity (cluster 5). None of the culture or sequencing batches contributed to the formation of any one of the final six clusters (Supplementary Fig. 4).

### Hierarchical clustering of EPA toxicological assays

Concentration at 50% maximum activity (AC_50_) values for all assays and all ToxCast Phase I chemicals were retrieved from the EPA Dashboard. We retained only those assays with data available for 99% of the chemicals and required that a given assay must have at least five active chemicals (AC_50_≤10 μM); 199 assays were retained. We then transformed the data values as described in Sipes *et al.*^64^ and performed hierarchical clustering to find the chemical groups with concordant activity on certain assays.

### Pathway analysis

To detect differentially regulated pathways, chemicals within each of the six clusters were separately compared with all other chemicals tested using GSA^65^, supplying a modified gene pathway file that contained all MSigDB C2 annotations as well as gene sets from published gene expression studies in human diseases and mouse models. Nearly all added gene sets were derived directly from the associated publication. Raw data derived from Durrenberger *et al.*^66^ were analysed by applying quantile normalization, and differential expression was detected using SAM using 500 permutations^67^. Some brain disease gene expression studies had no significantly differentially expressed genes (*q*<0.05), and hence could not be included in our analysis. For ‘BLALOCK_ALZHEIMERS_UP', a gene set included within MSigDB C2, we removed gene symbols that were not included in the RefSeq annotation. The Mod1 pathway (Gupta *et al.*^10^) was filtered to include genes with a KM1 score of 0.4 or greater, to reduce the number of genes in this pathway below 1,500 so it could be analysed by GSA. A given pathway (size between 10 and 1,500 genes) was considered if it achieved statistical significance (false discovery rate<0.1) for at least one cluster compared with all other chemicals (500 permutations). Expression of a single pathway was then summarized by taking the median expression value across all genes in that pathway for a given chemical. Once the entire data matrix was assembled, pathways were median-centred and hierarchically clustered, while keeping chemical ordering consistent with Fig. 2. Disease-relevant ontologies were then extracted and plotted separately; colour was applied based on the pathway enrichment score (blue indicates pathway downregulation and red indicates pathway upregulation), but only for the pathways achieving statistical significance (false discovery rate<0.1).

### Quantitative real-time PCR

Total RNA was extracted and purified using the RNeasy plus mini kit (Qiagen) following the manufacturer's instructions. RNA was quantified using Nanodrop 1000 (Thermo Scientific) and reverse transcribed using iScript Reverse Transcription Supermix (Bio-Rad). Quantitative real-time PCR analysis was performed in technical duplicates using SYBR Green PCR master mix (Applied Biosystems) in a 7500 Real-Time PCR instrument (Applied Biosystems). An amount of 10 ng of complementary DNA was used in each reaction. Fold change of expression of the target RNA in each treatment condition relative to the untreated sample were calculated by raising 2 to the power of −ΔΔCt. ΔΔCt was calculated by subtracting the ΔCt of the untreated sample to the ΔCt of each treatment condition. The ΔCt of each sample was calculated by subtracting the average Ct of *Gapdh* to the average Ct of the target RNA. The following primers were used at a final concentration of 0.5 μM:

*Rest* (F: 5′-GTGCGAACTCACACAGGAGA-3′, R: 5′-AAGAGGTTTAGGCCCGTTGT-3′); *Gsta4* (F: 5′-CGGCTGGAGTGGAGTTTGAG-3′, R: 5′-CCAAGGGTACTTGGCCGAAA-3′); *Gstm1* (F: 5′-CCGTGCAGACATTGTGGAGA-3′, R: 5′-CTGCTTCTCAAAGTCAGGGTTG-3′); *Hmox1* (F: 5′-AGGCTTTAAGCTGGTGATGGC-3′, R: 5′-GGGGCATAGACTGGGTTCTG-3′); and *Gapdh* (F: 5′-TATGACTCCACTCACGGCAAAT-3′, R: 5′-GGGTCTCGCTCCTGGAAGAT-3′).

### Fluorescence Immunohistochemistry

Mouse cortical neuron cultures were prepared identically as for live/dead and RNA-Seq experiments but at an equivalent density of 2.5 × 10^5^ cells per well in a 24-well plate containing precoated glass coverslips. At DIV 7, cells were treated with fenamidone at a concentration of 10 μM for 24 h. Coverslip-adherent cells were rinsed, paraformaldehyde fixed, blocked with 10% donkey serum and probed with the following cell-type-specific primary antibodies: guinea pig anti-NeuN (1:400, ABN90P, Millipore), goat anti-Gfap (1:1,200, ab53554, Abcam) and rabbit anti-Iba1 (1:400, 019-19741, Wako Pure Chemical Industries). Species-specific AlexaFluor-conjugated donkey secondary antibodies (1:200, Life Technologies) were then applied along with Nucblue (Life Technologies) to counterstain all nuclei. Coverslips were inverted into mounting substance (Fluorogel, 17985-10, Electron Microscopy Sciences) and imaged on a Zeiss LSM 710 laser scanning confocal microscope using a × 10 objective tiling scan of entire coverslips including three *z* planes to account for uneven coverslips. A researcher blind to the experimental conditions of images utilized maximal projected, spliced images to obtain particle counts for the total Nucblue-positive nuclei and the NeuN-positive neurons using particle analysis in ImageJ. Gfap- and Iba1-positive features containing clear Nucblue-positive nuclei were manually counted using the count function in Adobe Photoshop.

### Mitochondrial superoxide detection and live-cell imaging

Cultures were prepared on commercial poly-D-lysine-coated glass coverslips (GG-12-pdl, neuVitro) in 24-well plates at a density of 1.5 × 10^5^ cells per well. At DIV 7, cells were treated with chemical or an equivalent DMSO concentration in feeding medium for 2 h. Cells were then treated with MitoSOX Red (M36008, Life Technologies) mitochondrial superoxide indicator for 10 min at 37 °C. Medium containing the dye was aspirated, and the cells rinsed twice with 37 °C feeding medium and replaced with 37 °C artificial cerebral spinal fluid composed of 150 mM NaCl, 5 mM KCl, 1 mM MgCl_2_, 2 mM CaCl_2_, 10 mM HEPES and 10 mM dextrose (pH 7.3). Individual coverslips were transferred to a stage-top perfusion system mounted on an inverted Nikon Ti Eclipse microscope with constant flow of warmed artificial cerebral spinal fluid. Images (× 20) were collected using an Andora Clara charge-coupled device camera for brightfield and the Texas Red channel to ascertain the mitochondrial superoxide levels with the same exposure across all experiments. ImageJ software (NIH) was used to trace the soma of the cells in bright-field images as regions of interest (ROIs) using the polygon selection tool. ROIs were then superimposed on the fluorescent image and the total cell fluorescence was calculated per ROI to account for the size of the ROI and normalize to background fluorescent levels, as described^68^. Fluorescence intensity was then normalized to average corrected vehicle intensity. At least two non-overlapping fields from each coverslip were collected for each well. ROI tracing was performed by a researcher blind to the experimental condition. For select conditions, the proportion of cells showing altered soma morphology was manually calculated per image by the same experimenter performing ROI tracing.

### Tubulin polymerization assay

Chemicals were tested in a commercially available cell-free biochemical assay (BK011P, Cytoskeleton, Inc). Briefly, control and experimental solutions were prepared and loaded in duplicate into a warm (37 °C) 96-well plate. A solution of purified tubulin and a fluorescent reporter were added to the wells containing the warm substances and placed into a prewarmed (37 °C) Biotek Synergy HT microplate reader where fluorescence was measured every minute for 60 min. Equimolar doses of paclitaxel (included in kit) and vincristine (V8879, Sigma-Aldrich) were included as positive and negative modulators of tubulin polymerization, respectively.

### Chemical usage and approval data

Usage data were acquired from United States Geological Survey. Data are reported as total kilograms applied for each chemical across all US counties sampled per year (2000–2012) and a linear trend line was fit beginning in year 2000 or with the registration year (if after 2000). Registration dates for specific pesticide products were obtained from the National Pesticide Information Retrieval System (NPIRS; http://ppis.ceris.purdue.edu/). The NPIRS is a collection of pesticide-related databases, applications and websites under the administration of the Center for Environmental and Regulatory Information Systems at Purdue University, West Lafayette, Indiana. NPIRS obtains product information on a weekly basis from the EPA Pesticide Product Information System website. This Pesticide Product Information System information, including registered use sites and pests listed on the EPA stamped approved label, is disseminated through the NPIRS member and public websites, and is provided for informational purposes only. To ascertain which foodstuffs had the greatest amount of these chemicals, we ranked the five food commodities in descending order of maximum residue level detected using 2008–2012 data from the USDA Pesticide Data Program and the FDA Pesticide Program Residue Monitoring Program.

## Additional information

**Accession codes:** All RNA-seq data have been deposited in GEO under accession number GSE70249.

**How to cite this article:** Pearson, B. L. *et al.* Identification of chemicals that mimic transcriptional changes associated with autism, brain aging and neurodegeneration. *Nat. Commun.* 7:11173 doi: 10.1038/ncomms11173 (2016).

## Supplementary Material

Supplementary Information Supplementary Figures 1-12

Supplementary Data 1 Live/dead, vehicle-subtracted cytotoxicity results for all chemicals tested.

Supplementary Data 2 Brain disorders gene sets and genes. Differentially expressed gene lists were mined from the indicated publication or PubMed identifier (PMID).

Supplementary Data 3 Gene Set Analysis (GSA) results. Each tab corresponds to the significant gene sets hit for each of the six clusters (tested individually) relative to all other chemicals tested. Gene sets were considered significant at FDR < 0.1 and retained for subsequent clustering analyses (Supplementary Fig. 6) and visualization (Fig. 3). The Enrichment Score column is signed such that values < 0 refer to gene sets whose relative expression was lower in that given cluster than in all other chemicals.

Supplementary Data 4 Differentially expressed genes in cortical neuron cultures following treatment with 10 μM fenamidone or 0.1 μM pyraclostrobin for 24 h, n=4 sample replicates.
