# Supplementary material for: Identification of chemicals that mimic transcriptional changes associated with autism, brain aging and neurodegeneration
Source: Nat Commun. 2016 Mar 31;7:11173. doi: 10.1038/ncomms11173 (PMC4821887; doi:10.1038/ncomms11173)
Supplement: Supplementary Information — Supplementary Figures 1-12 [file ncomms11173-s1.pdf]

Supplementary Figure Legends

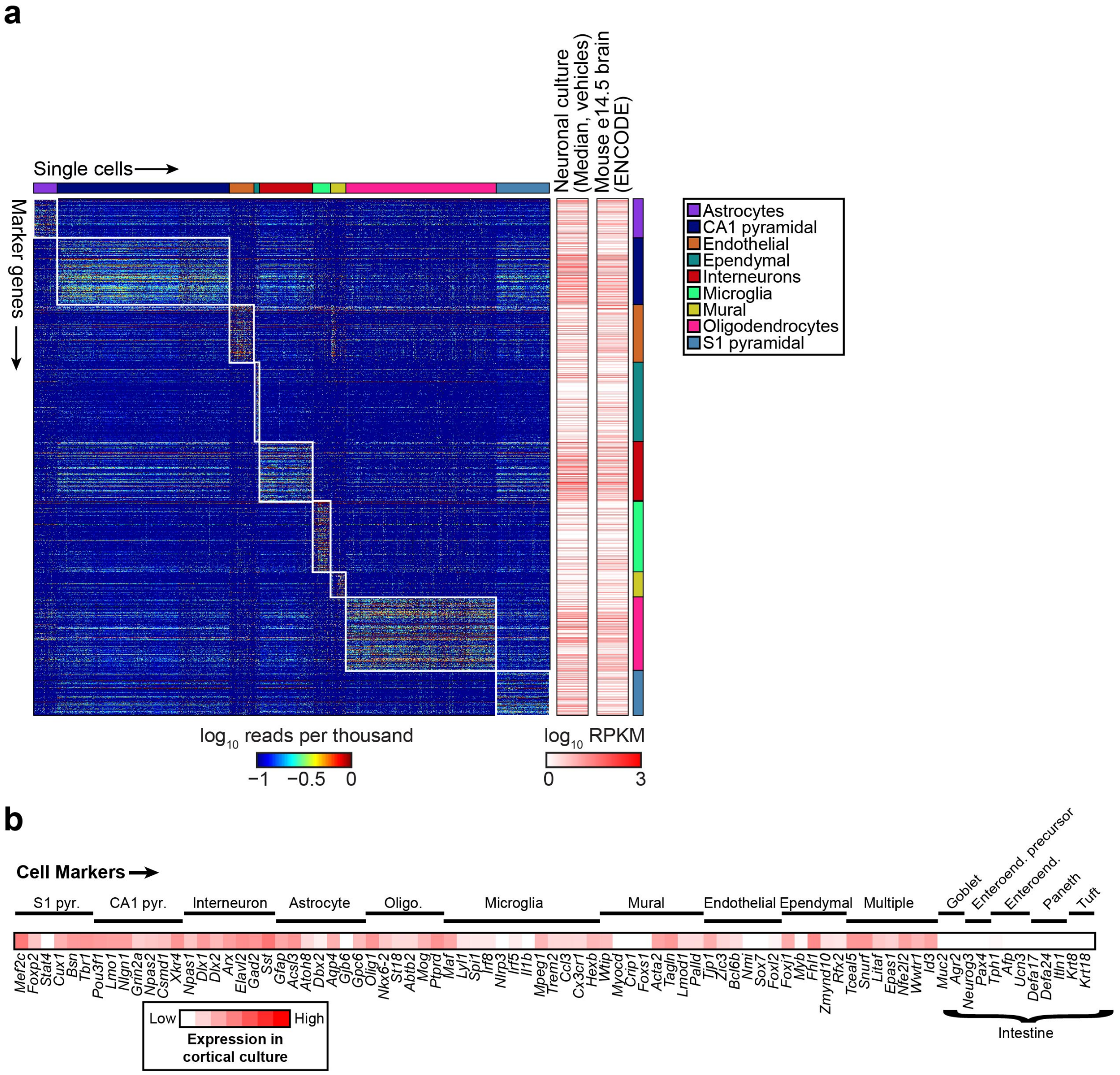

Supplementary Fig. 1. **Mouse cortical neuron cultures exhibit expression of marker genes from each of the major brain cell types.** (**a**) Expression of brain cell type marker genes in each of the single brain cells analyzed by Zeisel *et al,* as well as the expression of those marker genes in our culture system and in whole embryonic mouse brain (e14.5, ENCODE). (**b**) Vehicle-treated cortical cultures are enriched for brain cell-type marker genes but are not enriched for intestine cell-type marker genes.

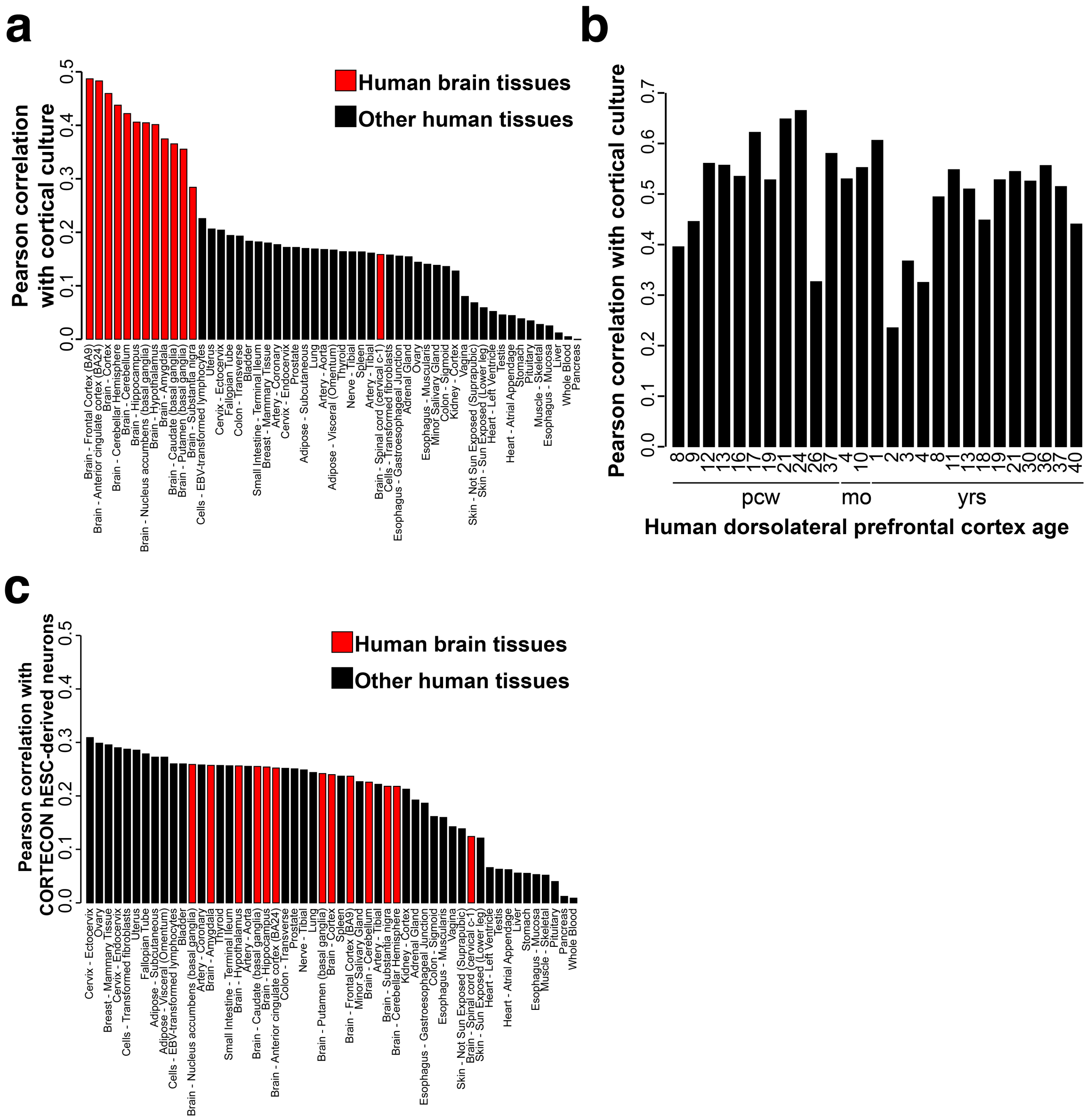

Supplementary Fig. 2. **Mouse cortical neuron cultures transcriptionally model the human brain.** (**a**) Cortical culture gene expression profiles correspond more closely to human brain tissues than other tissue types. (**b**) Cortical culture gene expression profiles correspond to human dorsolateral prefrontal cortex of many ages. (**c**) CORTECON hESC-derived neuron expression profiles correspond poorly to human brain regions relative to other human tissues.

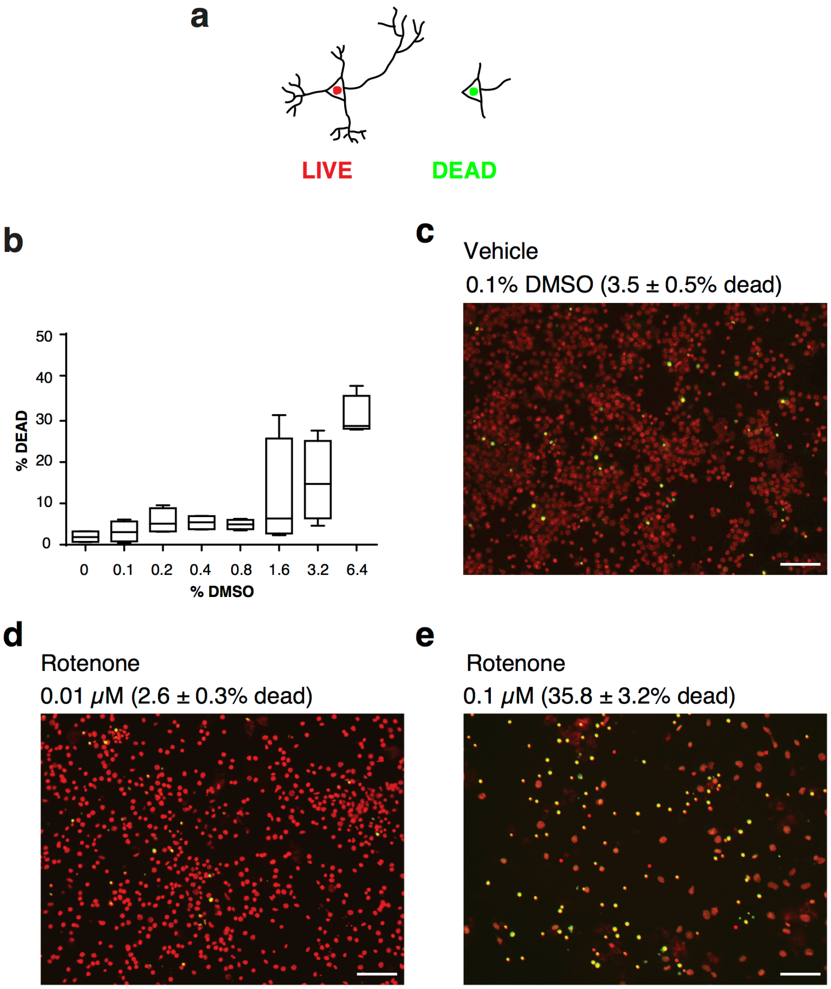

Supplementary Fig. 3. **Live/dead cytotoxicity assay.** (**a**) Cell permeable dye labeled all cells (red) while dead cells were labeled with membrane impermeable dye (green) in mouse cortical neuron cultures. (**b,c**) DMSO at 0.8% or lower caused little cell death. (**d**) Non-cytotoxic concentration of rotenone identified with live/dead assay (0.01 μM) and evaluated by RNA-seq. (**e**) Toxicity at higher rotenone concentration. Numbers represent mean percentage dead cells (vehicle subtracted) ± SEM across four replicates. Scale bars, 50 μm. Mean vehicle-subtracted values for all chemicals tested are provided in Supplementary Data 1.

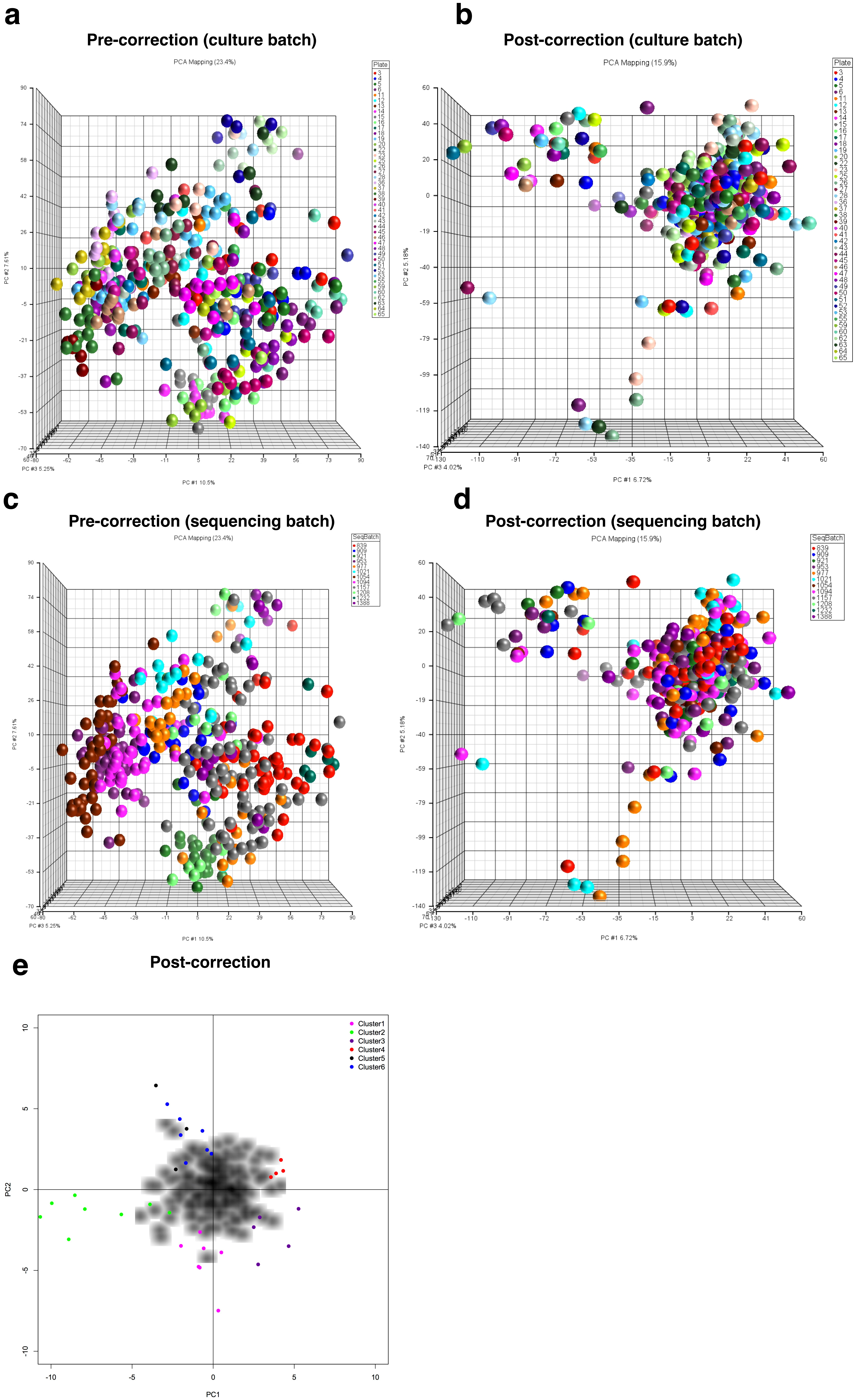
a
Pre-correction (culture batch)
PCA Mapping (23.4%)
Plate
PC #1 10.5%
PC #2 7.61%
PC #3 5.25%
b
Post-correction (culture batch)
PCA Mapping (15.9%)
Plate
PC #1 6.72%
PC #2 5.18%
PC #3 4.02%
c
Pre-correction (sequencing batch)
PCA Mapping (23.4%)
SeqBatch
839
909
921
953
977
1021
1054
1094
1157
1208
1232
1388
PC #1 10.5%
PC #2 7.61%
PC #3 5.25%
d
Post-correction (sequencing batch)
PCA Mapping (15.9%)
SeqBatch
PC #1 6.72%
PC #2 5.18%
PC #3 4.02%
e
Post-correction
Cluster1
Cluster2
Cluster3
Cluster4
Cluster5
Cluster6
PC1
PC2

Supplementary Fig. 4. **Effects and correction of cortical culture and sequencing batches.** (**a–b**) Batch correction by culture plate and (**c–d**) sequencing run removed non-specific culture and plate variability and (**e**) demonstrated intact, post-normalization cluster grouping.

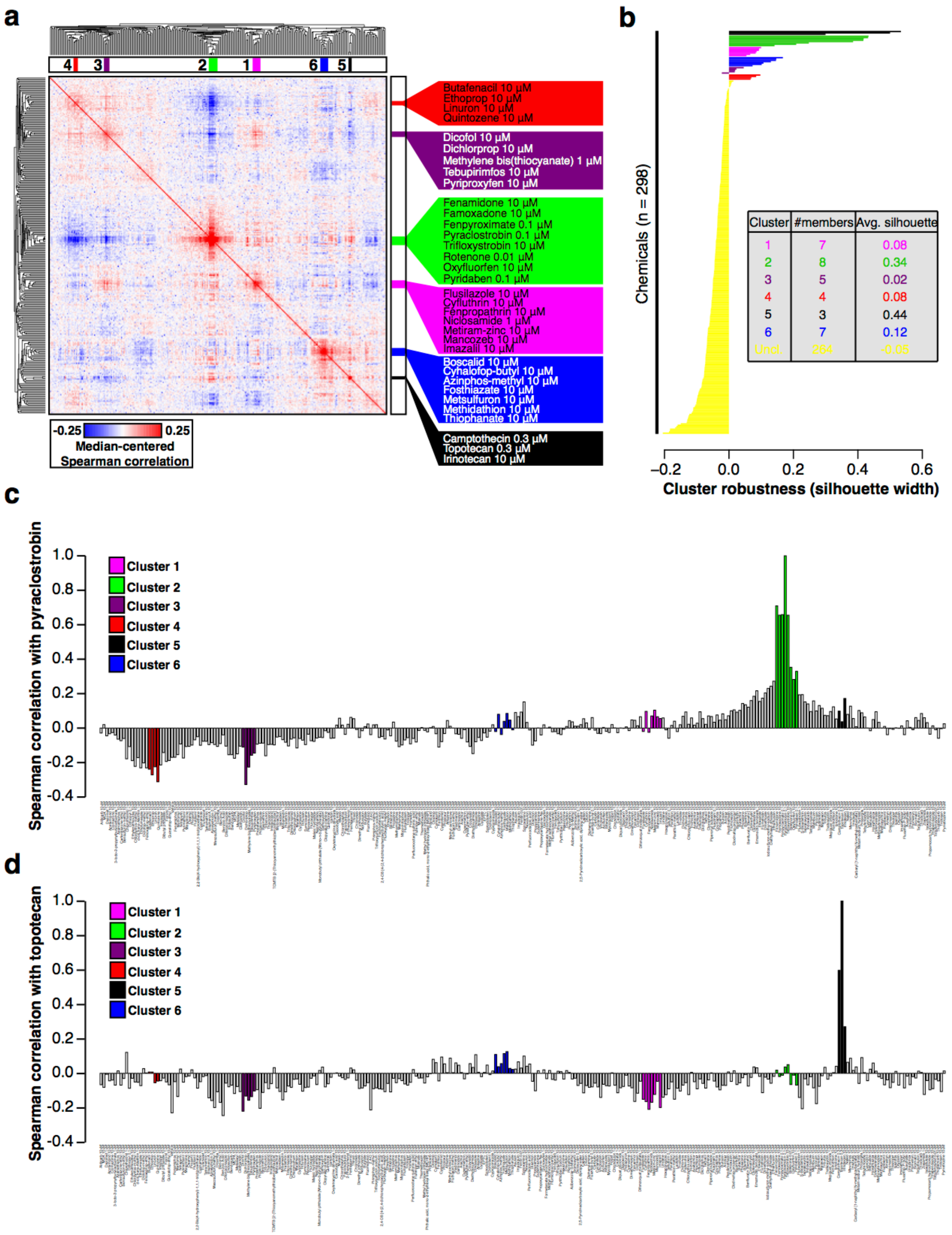

Supplementary Fig. 5. **Criteria used to establish cluster membership.** (**a**) Expression data of each chemical was compared pairwise by plotting the median-centered Spearman correlation. A given cluster had to have at least three members with the minimum

pairwise Spearman correlation coefficient exceeding 0.2 and a positive silhouette width. (**b**) Positive silhouette widths of chemicals falling within defined clusters illustrates robust cluster membership. (**c–d**) Examples of transcriptional similarity within Cluster 2 (**c**) and Cluster 5 (**d**).

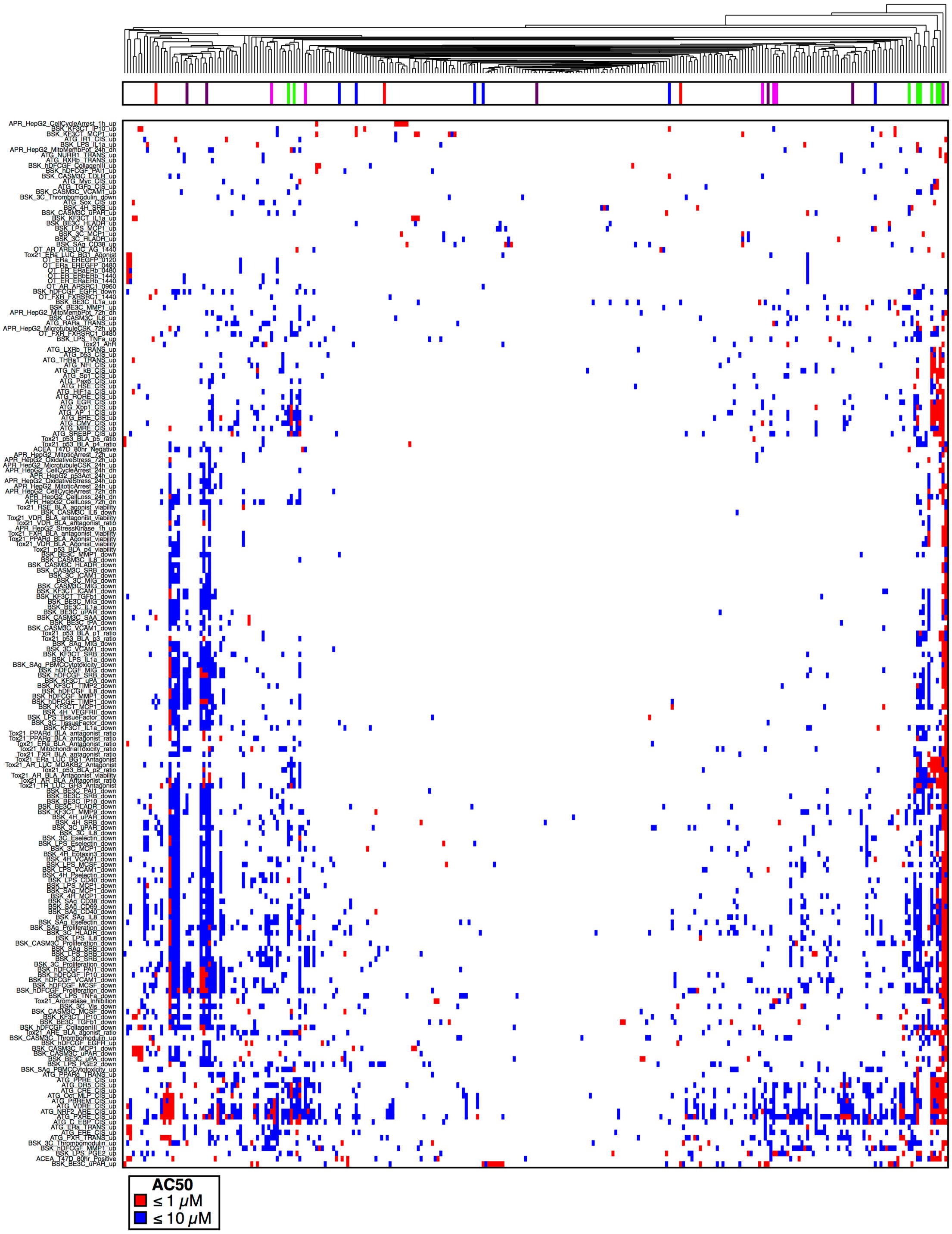

Supplemental Fig. 6. **Analysis of existing *in vitro* assay data with ToxCast chemicals.** Hierarchical clustering of published assay data compared to cluster groups identified by transcriptional profiling (color coded according to Fig. 2 in rectangle oriented below

cladogram). Heatmap coloring is set according to chemical-assay potency indicated in small legend below heatmap.

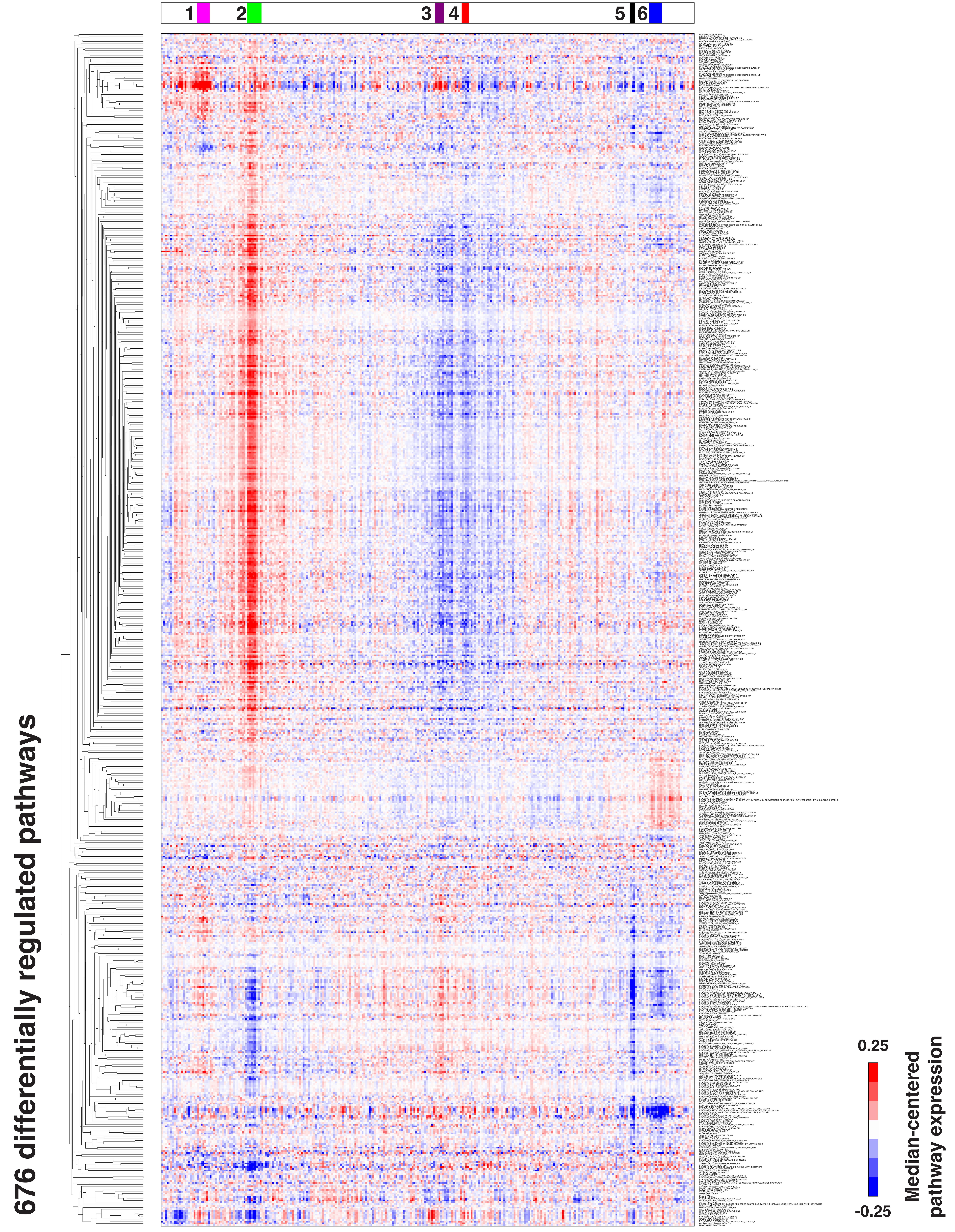
1
2
3
4
5
6
676 differentially regulated pathways
0.25
-0.25
Median-centered pathway expression

Supplementary Fig. 7. **Differential gene set enrichment across the six clusters.** Expression data of chemicals within each of the six clusters were separately compared to all other chemicals using GSA. A given pathway (size between 10 and 1,500 genes) was considered significant if the false discovery rate (FDR) was less than 0.1 for at least one cluster compared to all other chemicals (500 permutations). Expression of a single pathway was summarized by taking the median expression value across all genes in that pathway for a given chemical. Pathway expression values were then median-centered and hierarchically clustered, while keeping chemical ordering consistent with Fig. 2. Differentially enriched gene sets per cluster are included in Supplementary Data 3.

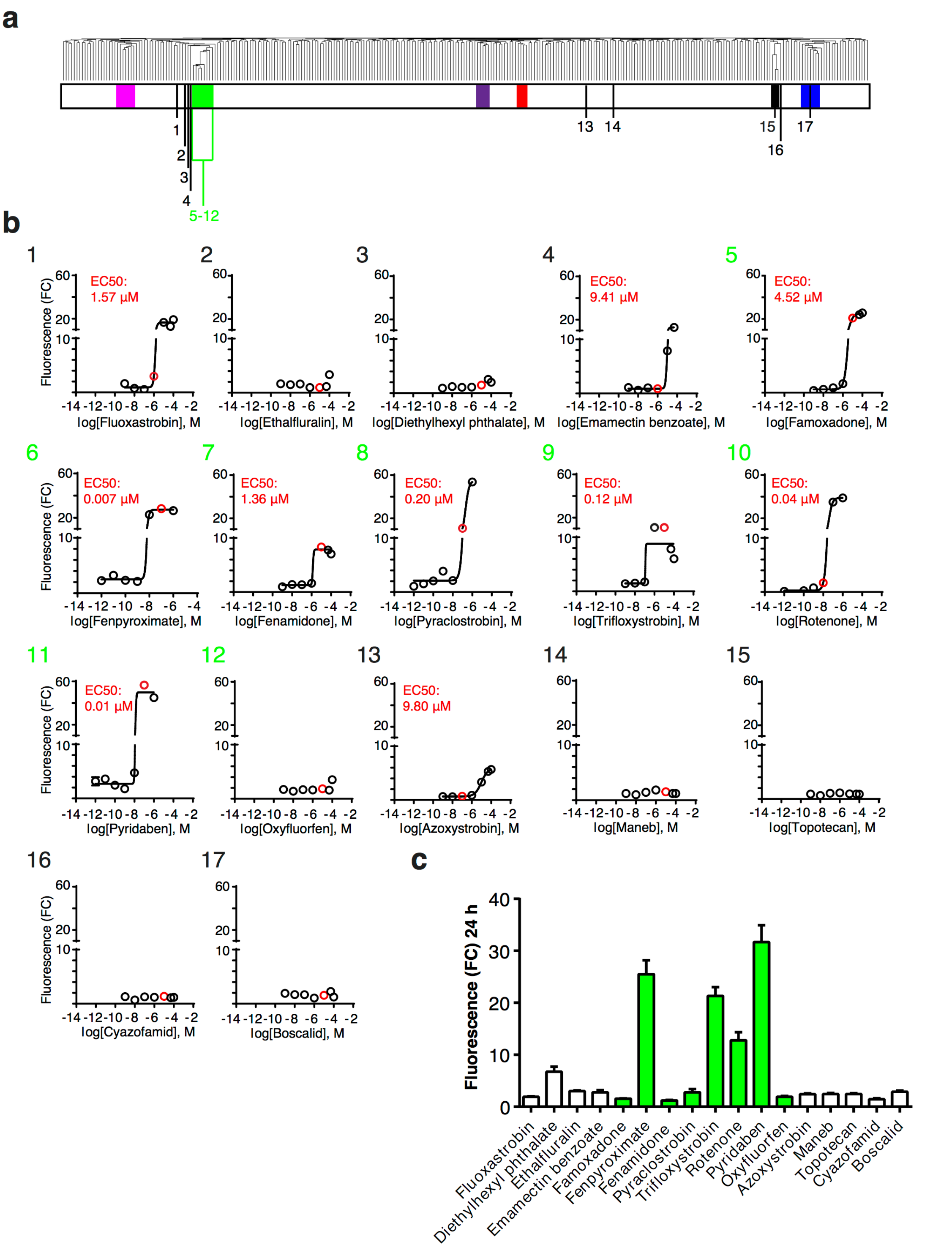

Supplementary Fig. 8. **Cluster 2 chemicals produce dose-dependent superoxide.** (**a**) Chemical hierarchy, ordered identically to Fig. 2, with the indicated chemicals (1-17) tested for (**b**) superoxide production (2 h chemical treatment followed by MitoSOX live

cell imaging). RNA-seq doses are indicated with red circles. (**c**) O_2^- production after treating with the indicated chemicals for 24 h at the sequencing dose followed by MitoSOX imaging.

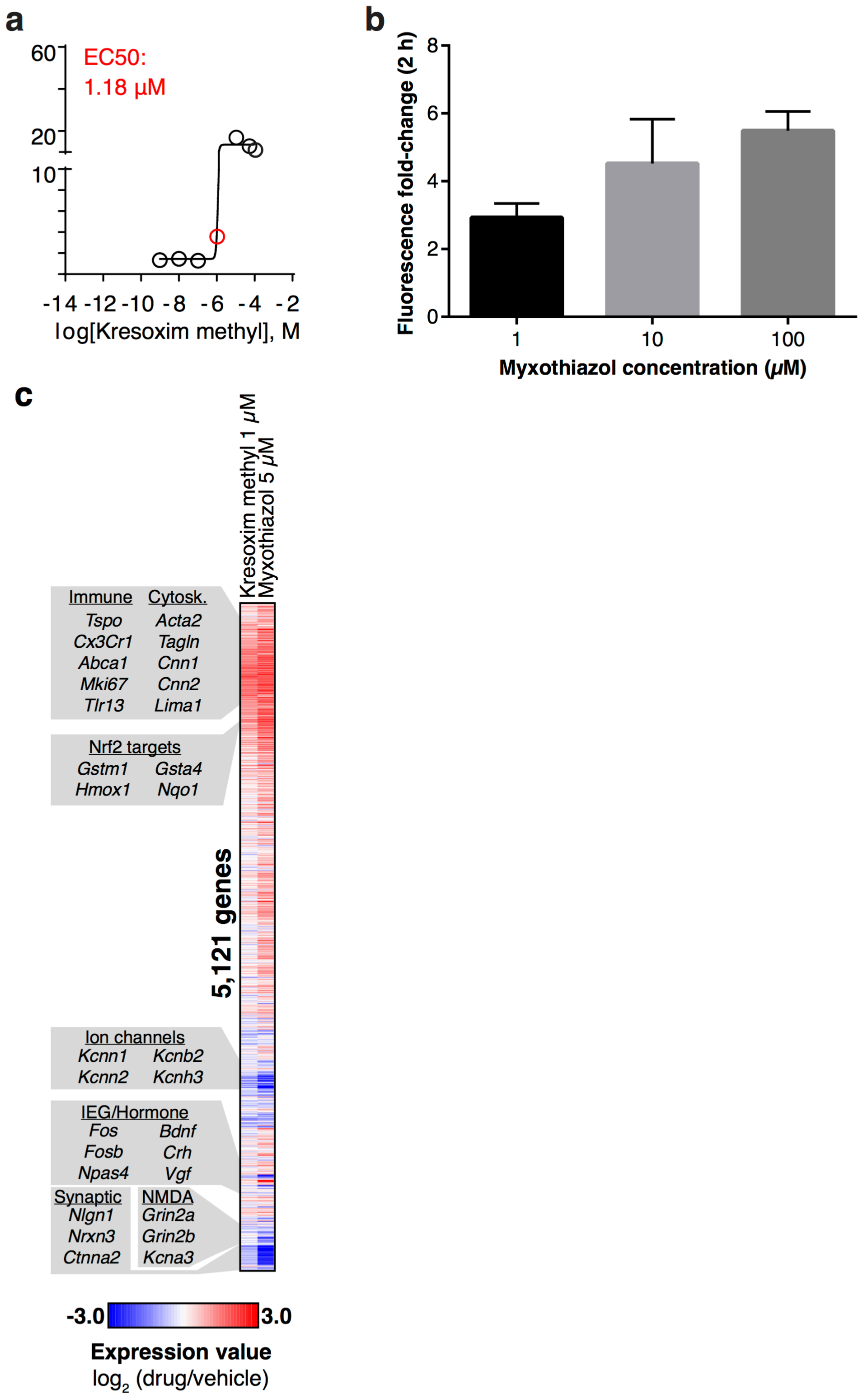

Supplementary Fig. 9. **Effects of Cluster 2 chemicals can be phenocopied with additional mitochondrial poisons.** (**a**) Kresoxim methyl and (**b**) myxothiazol induce O_2^- production in a dose-dependent manner. When added to cortical cultures for 24

hours, kresoxim methyl (1 μM) and myxothiazol (5 μM) elicited corresponding gene expression (RNA-seq) effects with Cluster 2 chemicals. Gene order is identical to Fig. 2.

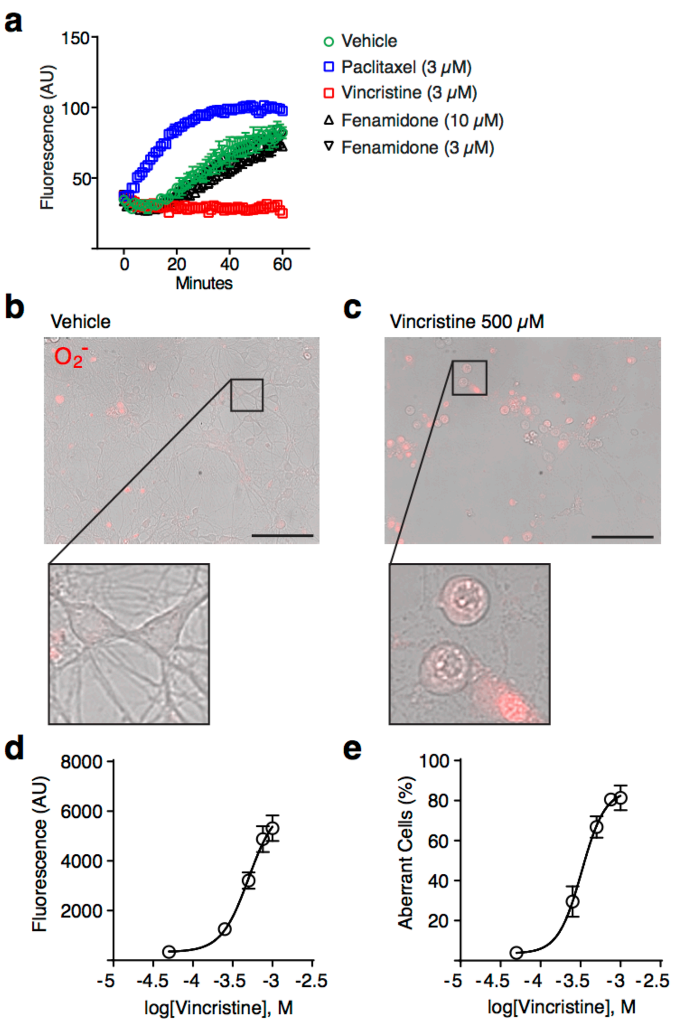

Supplementary Fig. 10. **A microtubule destabilizer (vincristine) promotes O_2^- production and aberrant cell morphology.** (**a**) Vincristine, but not fenamidone, inhibited purified tubulin polymerization in a cell-free biochemical assay. Paclitaxel (positive control) promoted tubulin polymerization. (**b-c**) Vincristine (500 µM, 2 h) induced mitochondrial superoxide (O_2^-; MitoSOX fluorescent indicator) generation and altered cell morphology in cortical neurons. Scale bars, 100 µm. (**d**) O_2^- production and (**e**) aberrant cell morphology were dose-dependent. n=60-119 cells/coverslip, across two coverslips per concentration.

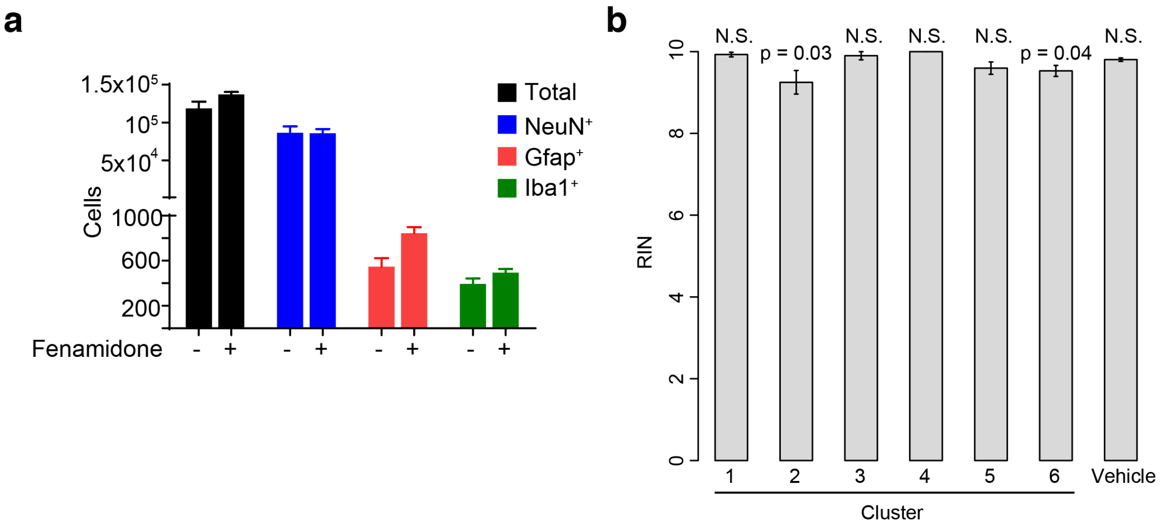

Supplementary Fig. 11. **Cluster 2 chemicals not linked to altered cellular composition or RNA quality.** (**a**) The number of NeuN, Gfap, or Iba1 positive cells did not significantly change after treatment with 10 µM fenamidone for 24 hours, relative to vehicle. n=4 cultures/condition. (**b**) RNA integrity numbers (RIN) for all chemicals in each of the six clusters as well as all vehicle replicates. Bars are mean ± SEM. Statistical significance was assessed using a one-way t-test comparing the chemicals in a given cluster to vehicle control.

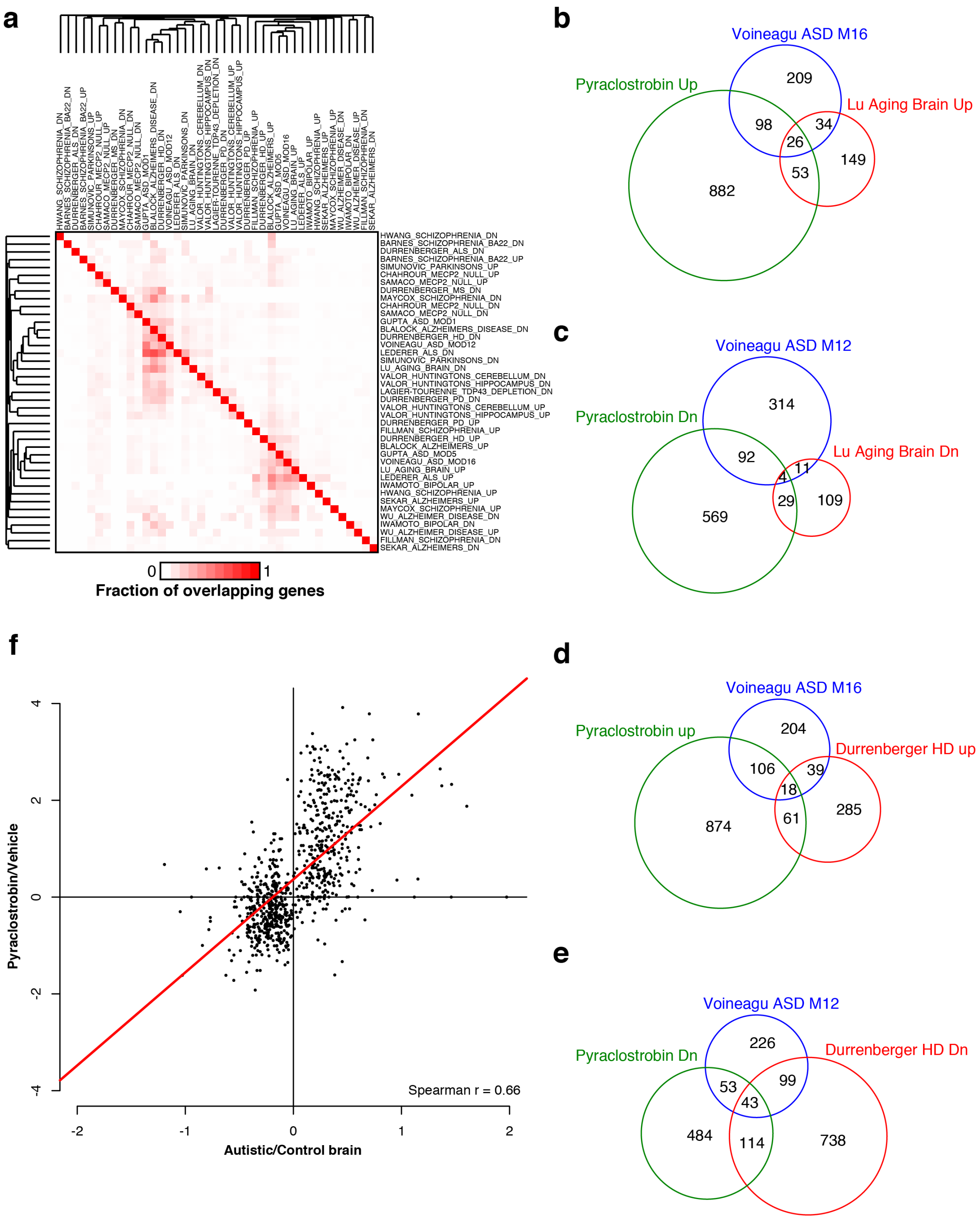

Supplementary Fig. 12. **Overlap of brain disease gene sets.** (**a**) Pairwise degree of overlap (red intensity) between brain disease datasets (from Fig. 3) shows correspondence in up- and down-regulated genes across diseases of diverse pathology. (**b–e**) Degree of overlap and directionality of overlapping transcripts included from

pyraclostrobin treated neurons, ASD, Huntington's (HD) and aging post-mortem brain.

(**f**) Comparison of the change in gene expression (fold-change) elicited by pyraclostrobin treatment in neurons to changes (fold-change) seen in ASD brain compared to control brain demonstrates consistent direction and magnitude of gene expression changes.
